# Anti-Migratory Effects of 4′-Geranyloxyferulic Acid on LPS-Stimulated U937 and HCT116 Cells via MMP-9 Down-Regulation: Involvement of ROS/ERK Signaling Pathway

**DOI:** 10.3390/antiox9060470

**Published:** 2020-06-01

**Authors:** Sara Franceschelli, Daniela Maria Pia Gatta, Alessio Ferrone, Giulia Mezza, Lorenza Speranza, Mirko Pesce, Alfredo Grilli, Marialucia Gallorini, Viviana di Giacomo, Barbara Ghinassi, Serena Fiorito, Salvatore Genovese, Emanuela Ricciotti, Mario Felaco, Antonia Patruno

**Affiliations:** 1 Department of Medicine and Science of Aging, University “G. D’Annunzio”, 66100 Chieti-Pescara, Italy; sara.franceschelli@unich.it (S.F.); alessioferrone@yahoo.it (A.F.); lorenza.speranza@unich.it (L.S.); mirko.pesce@unich.it (M.P.); barbara.ghinassi@unich.it (B.G.); mario.felaco@unich.it (M.F.); 2 Department of Oncology, ASL2, SS Annunziata Hospital, 66100 Chieti-Pescara, Italy; dottdanielagatta@gmail.com; 3 Department of Pharmacy, University “G. D’Annunzio”, 66100 Chieti-Pescara, Italy; giulia.mezza@studenti.unich.it (G.M.); marialucia.gallorini@unich.it (M.G.); Viviana.digiacomo@unich.it (V.d.G.); serena.fiorito@unich.it (S.F.); salvatore.genovese@unich.it (S.G.); 4 Department of Medical, Oral and Biotechnological Sciences, University “G. D’Annunzio”, 66100 Chieti-Pescara, Italy; alfredo.grilli@unich.it; 5 Department of Systems Pharmacology and Translational Therapeutics, University of Pennsylvania, Philadelphia, PA 19104, USA; emanuela@pennmedicine.upenn.edu

**Keywords:** proliferation, migration, apoptosis, senescence, metalloproteinase, colorectal cancer cells, lymphocytic histiocytoma cells, ROS, p38, pERK, 4′-geranyloxyferulic acid

## Abstract

Matrix metalloproteinases (MMPs) play a crucial role in tumor angiogenesis, and metastasis. 4′-geranyloxyferulic acid (GOFA) has anti-tumor and anti-inflammatory proprieties. Herein, we aimed to determine whether this compound affects cell survival, invasion, and migration through reactive oxygen species (ROS)-mediated MMPs activation of extracellular signal-regulated kinases (ERKs) and p38 signaling in lymphocytic histiocytoma (U937) and colorectal cancer (HCT116) cells. We observed that lipopolysaccharide (LPS) stimulated U937 and HCT116 cells presented abnormal cell proliferation and increased metalloproteinase (MMP-9) activity and expression. Non-cytotoxic doses of GOFA blunted matrix invasive potential by reducing LPS-induced MMP-9 expression and cell migration via inhibiting ROS/ ERK pathway. GOFA also attenuated apoptosis and cell senescence. Our findings indicate that GOFA, inhibiting cancer cell proliferation and migration, could be therapeutically beneficial to prevent tumor metastasis.

## 1. Introduction

Matrix metalloproteinases (MMPs) are a family of enzymes, Zn^2+^-dependent endopeptidases, implicated in tissue modeling through their skill to hydrolyze the protein components of the extracellular matrix (ECM) [1]. This family of enzymes are classified according to their substrate specificity (for example, matrilysins, stromelysins, gelatinases, collagenases, and membrane-type MMPs) and named in the chronological order of discovery [2].

MMP-2 (gelatinase A) and MMP-9 (gelatinase B), also named type IV collagenases, play an essential role in the chronic inflammatory process. In addition, MMP-2 is constitutively and ubiquitously expressed in nearly all cells of the body, whereas MMP-9 is a cytokine-inducible enzyme particularly expressed by immune cells during the inflammation process [3].

Inflammation is a common and physiological response to harmful stimuli and nowadays is well known to be linked with the pathogenesis of several acute and chronic diseases [4]. The inflammatory response, which is responsible to cell proliferation and invasion, involves several secreted molecules such as cytokines, chemokines, and various signaling molecules [5,6]. In this context, the production of reactive oxygen species (ROS) is central to the evolution of inflammation and for the progression of several basic biological processes, including cellular proliferation and differentiation. ROS are produced by cells that are involved in the host-defense response by the oxidation of key cellular signaling proteins [7,8].

The ROS also play a considerable role as signaling molecules which regulate the expression of many genes, including MMPs [9].

The breakdown of the extracellular matrix (ECM) is a crucial issue of several biological processes such as wound healing, immune cell infiltration, angiogenesis, embryogenesis, and other physiological processes [10]. Most MMPs are secreted as zymogens that become triggered by proteases and/or ROS or other MMPs [11]. MMPs play an important role in the pathogenesis of inflammatory diseases with considerable tissue damage, such as rheumatoid arthritis, chronic cutaneous ulcerations, inflammatory bowel disease (IBD) as well as in cancer progression [12]. The downregulation of expression and activity of MMPs is under stringent control in physiological situations, whereas the upregulation of the activity of MMPs is frequent in pathological conditions. During metastasis, many regulatory events happen, with the involvement of specific molecules. The main metastatic processes include migration and invasion, circulation and adhesion, and colonization. In the early stage, the alteration of ECM, responsible for cell–cell contact induces metastasis and migration. Thus, MMPs contribute to tumor cells’ ability to invade and metastasize [13]. For these reasons, MMPs represent an attractive therapeutic target for the treatment of inflammatory diseases, such as cancer.

**Figure 1:**
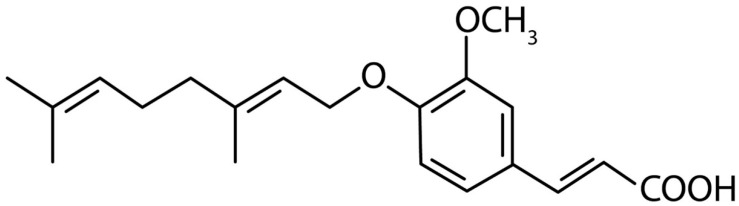
Chemical structures of 3-(4′-geranyloxy-3′-methoxyphenil)-2-trans propenoic acid (GOFA).

Indeed, many studies have confirmed that chronic inflammation increases the risk of cancer, promotes tumor progression, and supports metastatic spread. Inhibition of cancer cell migration, invasion and adhesion and/or regulators of expression may be a real strategy for restraining metastasis [14]. In recent years, several phenolic compounds (such as ferulic acid derivatives), have been discovered to present a range of pharmacological and biological effects, like anti-hyperlipidemia, anti-atherogenic, anti-diabetes, anti-tumor, anti-inflammatory and antioxidant [15]. 3-(4′-Geranyloxy-3′-methoxyphenyl)-2-trans propenoic acid denominated 4′-geranyloxyferulic acid (GOFA, Figure 1) is an oxyprenylated ferulic acid derivative that was extracted for the first time in 1966 from the root and bark of *Acronychia baueri* Schott (Fam. Rutaceae). In recent years, the pharmacological properties of this compound have begun to be described and assessed, showing anti-tumor and anti-inflammatory proprieties [16].

Inflammation plays a multifarious role in cancer development. Lipopolysaccharide (LPS) is a glycolipid of the outer membrane of Gram-negative bacteria, responsible for increased production of proinflammatory cytokines [17].

Reports have suggested that LPS acts not only on immune cells but also on some types of epithelial cells including cancer cells [18].

During cellular activation, LPS, complexes with LPS-binding protein (LBP) and CD14 to activate intracellular transduction signaling through Toll-like receptor 4 (TLR4) [19]. TLR4, which is expressed in many human cancer cell lines, plays an important role in linking LPS to inflammation and cancer invasion and progression [20].

Based on this premise, we investigated the effect of GOFA on lymphocytic histiocytoma (U937) cells and colorectal cancer (HCT116) cells migration induced by LPS. In addition, we explored the possible molecular mechanisms involved in the process.

## 2. Materials and Methods

### 2.1. Cell Culture

Human monocytes cell line U937 (ATCC^®^ CRL-1593.2™) and human colorectal carcinoma cell line HCT 116 (ATCC^®^ CCL-247™) were cultured at a density of 10^6^ cells/mL in RPMI 1640 medium (Sigma-Aldrich, MA, USA) and in McCoy’s 5A modified medium (Sigma-Aldrich, MA, USA) respectively. The cells were cultured as reported previously [21,22].

The cell viability, determined by trypan blue exclusion, was >99%. Cells were seeded onto six-well tissue culture plates and incubated overnight at 37 °C in a humidified atmosphere of 5% CO_2_. More than 98% of cells were viable, as determined by trypan blue dye exclusion at the starting point of the culture, and more than 90% were viable at the time of cell collection.

For the experimental set point, the U937 cell line was treated with 1, 10, 25, 50 and 100 µM of GOFA, and the HCT116 cell line was treated with 0.1, 1, 10, 50 and 100 µM of GOFA. GOFA was synthesized in our laboratories as previously reported [23]. In some experiments, cells were treated with LPS (10 µg/mL, Sigma, St Louis, MO, USA), extracellular signal-regulated kinase (ERK)1/2 inhibitor (5 µM) (PD980559, Calbiochem, San Diego, CA, USA), p38 inhibitor (30 µM) (SB203580, Calbiochem, San Diego, CA, USA) and/or N-Acetyl-L-cysteine (3 mM, NAC, A7250 Sigma, St Louis, MO, USA). GOFA was added to the culture medium 30 min before the stimulation with LPS, while the other compounds were added 60 min before LPS.

### 2.2. MTT Assay for Cell Viability and Cytotoxicity 

The MTT assay was used to assess cell viability and cytotoxicity of GOFA on both cell lines. Briefly, the U937 and HCT116 cells were seeded on 96-well plates at a density of 8 × 10^3^ cells/well, and cultured and treated according to the method described above [24]. The MTT assay was performed in experiments with U937 or HCT116 cells treated with different concentrations of GOFA as reported above, with and without LPS (10 µg/mL Sigma, St Louis, MO, USA). The MTT (20 µL; 0.5 mg/mL) and the culture medium (200 µL) were added to each well and, to dissolve the formazan that had formed, and the plates were incubated at 37 °C for 4 h. When this solution (220 µL) was removed, 150 µL of DMSO was added to each well and the reduced MTT was quantized at a wavelength of 570 nm on an ELISA reader (Bio-Rad, Hercules, CA, USA). The cell viability percentage was calculated using to the equation below:

% = (Absorbance of treated cells/Absorbance of control cells) × 100(1)

### 2.3. Cell Cycle Analysis

Approximately 0.5 × 10^6^ cells per experimental state were harvested, fixed in 70% cold ethanol, and kept overnight at 4 °C. Cells were then re-suspended in 20 µg/mL propidium iodide (PI) and 100 μg/mL RNase, final concentrations. Cell cycle profiles (10,000 cells) were evaluated with a FC500 flow cytometer with the FL3 detector in a linear mode using the CXP software (Beckmann Coulter, Miami, FL, USA). Data were investigated with the MultiCycle software (Phoenix Flow Systems, San Diego, CA, USA).

### 2.4. Migration Assay for U937 Cells

The quantitative migration assay was performed using a modified Boyden chamber (Transwell^®^, Corning, NY, USA) with a pore polycarbonate filter (8.0-µm) inserted in a 24-well plate. After having counted the cells, 0.5 mL of the starved cell suspension was seeded on the upper chamber (2.5 × 10^3^) in different culture conditions: untreated cultures (Ctrl), GOFA, GOFA+LPS. The bottom chamber was filled with 0.6 mL of complete growth medium. Following 24 h incubation, cells from both the upper and the bottom chamber were harvested separately, and the number of migrated cells was assessed by flow cytometry (CytoFLEX, Beckman Coulter, CA, USA). Before running samples, morphological parameters (Side Scatter/Forward Scatter, SSC/FSC) and a defined acquisition time (1 min) were set up. Data were expressed as the number of cells refracting the laser emission through the CytExpert Software (Beckman Coulter, CA, USA).

### 2.5. Scratch Assay

The scratch assay is a well-developed method to investigate the HCT116 cell migration in vitro. The cells were seeded at a density of about 1 × 10^6^ cells/well (10^4^ cells/cm^2^, in 6-well plates) in complete medium at 37 °C and 5% CO_2_ (*v*/*v*), and grown for 24 h to allow them to reach about 90% confluence. Scratches were created mechanically with a sterile pipette tip (Ø = 0.1 mm) on cell monolayer. We were careful to produce uniformly sized wounds of approximately 0.5 mm, and debris was removed from the culture and cells were then cultured with fresh medium. The size of the gap at a selected position was measured using the cell Sense Imaging software (Olympus, Hamburg, Germany) at the starting point of the experiment (0 h), after 24 h and up to 48 h. The ratio of the gap size at certain time points to the gap size at 0 h was calculated and replicates were averaged. Scratch closure was assessed every 2 h using an inverted microscope (Leica DM IL D-35578 Wetzlar, Germany) at a magnification of x10 and photographed with a Colour View II digital camera to measuring the remaining cell-free area in triplicates wells. The results were expressed as percentage of the cell-free area at T 24 h or T 48 h compared to T_0_ and represented as mean ± SD of 3 independent experiments.

### 2.6. Analysis of MMP-9 Activity by Gelatin Zymography

Gel zymography was performed as previously described [25]. Cellular supernatants were collected, and protein content was determined by Bradford assay (Bio-Rad, Hercules, CA, USA). Samples were loaded and subjected to 8.5% SDS-PAGE (Bio-Rad) containing porcine gelatin at 4.5 mg/mL (Sigma-Aldrich, St Louis, MO, USA) in non-denaturing, non-reducing conditions. The MMP-9 band density quantification was performed using Gel Doc 1000 (Bio-Rad).

### 2.7. mRNA Extraction and qRT-PCR Analysis

Extraction of total RNA from cells was performed using TRIzol reagent (Invitrogen, Carlsbad, CA, USA), following to the manufacturer’s protocol. Total RNA concentration was measured at 260 nm using a Bio-Photometer (Eppendorf AG, Hamburg, Germany). RNA samples were preserved frozen at −80 °C until use. A quantitative RT-PCR assay was performed in an Eppendorf Mastercycler ep realplex (Eppendorf AG) as described previously [26]. Preliminary PCR reactions were run to optimize the concentration and ratio of each primer set. For all cDNA templates, 2 μL was used in a 20 μL real-time PCR amplification system of SYBR Green Real Master Mix Kit following the manufacturer’s protocol. Primers targeting MMP-9 and 18S rRNA were designed using GeneWorks software (IntelliGenetix, Inc., World Wide Corporation, Mountain View, CA, USA) (MMP-9 forward: 5′-AGTGGCACCACCACAACAT-3′; reverse: 5′ CCTGGGTGTAGAGTCTCTCG-3′; 18S forward: 5′-CTTTGCCATCACTGCCATTAAG-3′ and reverse: 5′-TCCATCCTTTACATCCTTCTGTC-3′). The fluorescence intensity of the double strand-specific SYBR Green, reflecting the amount of formed PCR product, was monitored at the end of each elongation step. Melting curve analysis was performed to confirm the purity of the PCR products. Variations in MMP-9 gene expression were analyzed using the (ΔΔCt) method (relative expression =2Δ^−CT^, where ΔCT = C_T(MMP9)_ − C_T(18S)_) with a threshold cycle value of 0.2 normalized to 18S rRNA. Predicted cycle threshold values were exported directly into Excel worksheets for analysis. Relative changes in gene expression were reported as a calibrator-normalized ratio relative to the calibrator cDNA (control value = 1) prepared in parallel with the experimental cDNAs. The experiments were repeated twice with consistent results and data are presented as means ± SD from biological triplicates.

### 2.8. Western Blot

Western blot analysis was performed as reported previously [27]. U937 and HCT-116 cells were collected and lysed in RIPA buffer. Total protein extracts were separated on a 4–12% NuPAGE gradient gel (Gibco Invitrogen, Paisley, UK). Blots were probed and incubated overnight with primary antibodies for p-p38 (Tyr182, sc-101759), p-ERK (Thr202, sc-101760), and β-actin (sc-47778) (Santa Cruz Biotechnology, Santa Cruz, CA, USA). The Super Signal Ultra chemiluminescence detection reagents (Pierce Biotechnology, Rockford, IL, USA) were used to detect the protein expression. A gel analysis software package (Gel Doc 1000; Bio-Rad) was employed to analyze the blot images.

### 2.9. ROS Production Analysis 

ROS measurement was performed as described previously [28]. Cells (5 × 10^5^) were resuspended with carboxy-2′,7′-dichloro-dihydro-fluorescein diacetate (DCFH-DA Sigma, St Louis, MO, USA) probe and incubated at 37 °C for 30 min in the dark. The fluorescence was determined after 24 h of treatment measuring the absorbance at 485 (excitation) and 535 nm (emission) wavelengths using a dark microplate reader (Molecular Devices Spectra MAX Gemini X).

### 2.10. Cell Senescence Assay

Senescence-associated β-galactosidase (SA-β-Gal) was performed using the fluorescent cellular senescence assay kit from Cell Biolabs (San Diego, CA, USA) and following the manufacturer’s recommended protocol. Briefly, cells were washed with cold PBS and lysed. Lysates were centrifuged and supernatant was collected. After transfer to fluorescence 96-well plates, the lysates were incubated with the assay buffer for 1 h at 37 °C. The reaction was stopped, and the fluorescence was measured at 360 (excitation) and 465 nm (emission) on a dark microplate reader (Molecular Devices Spectra MAX Gemini X).

### 2.11. Annexin V/PI Detection of Apoptotic and Necrotic Cells in Flow Cytometry

To assess apoptosis, a commercial Annexin V-FITC/PI kit (Bender MedSystems GmbH, Vienna, Austria) was used following the manufacturer’s protocols, as previously reported [29]. Briefly, the cells were gently re-suspended in binding buffer and incubated for 10 min with Annexin V-FITC at room temperature in the dark. Samples were then washed and supra-vitally stained with PI (5 μg/mL). The analysis was performed with a FC500 flow cytometer with the FL1 and FL3 detector in a log mode using CXP analysis software (Beckmann Coulter). For each sample, 10,000–20,000 events were saved. Viable cells were Annexin V^neg^/PI^neg^ (unlabelled), early apoptotic cells were Annexin V^pos^/PI^neg^, late apoptotic and necrotic cells were Annexin V^pos^/PI^pos^ and Annexin V^neg^/PI^pos^, respectively.

### 2.12. Caspase 3 Assay 

The fluorogenic substrate Ac-DEVD-pNA (Ac-Asp-Glu-Val-Asp-7-amino-4-methylcoumarin; Enzo Life Sciences LTD, Exeter, UK) was used to perform Caspase-3 (EC 3.4.22.56) activity. First, the cells were centrifuged (1000× *g*, 5 min, 4 °C) and then lysed using a lysis buffer (50 mM Tris pH 7.5, 150 mM NaCl, 5 mM ethylenediaminetetra-acetatedihydrate (EDTA), and 0.2% Nonidet P-40). After centrifugation (17,000× *g*, 10 min, 4 °C) the reaction buffer containing 4-(2-hydroxyethyl) piperazine-1-ethanesulfonic acid (HEPES)) (10 mM, pH 7.5), NaCl (50 mM), MgCl_2_ (5 mM), dithiothreitol (DTT) (2.5 mM), and EDTA (1 mM) was added. Finally, the samples were incubated with the substrate (50 µM) and the fluorescence (substrate turnover) was evaluated measuring, in a 96-well microplate reader (model 550; Bio-Rad Laboratories, Inc., Hercules, CA, USA), the excitation and emission at 360 and 460 nm, respectively. The rate of substrate hydrolysis was monitored at 37 °C.

### 2.13. Statistical Analysis

All results were expressed as mean ± SD, for each assessment performed at least in triplicate. One-way ANOVA was performed to compare means between groups. A probability of null hypothesis of 5% (*p* < 0.05) was considered as statistically significant. Data were analyzed with SPSS v19.0.1 (SPSS) for Windows.

## 3. Results

### 3.1. Cell Viability

**Figure 2:**
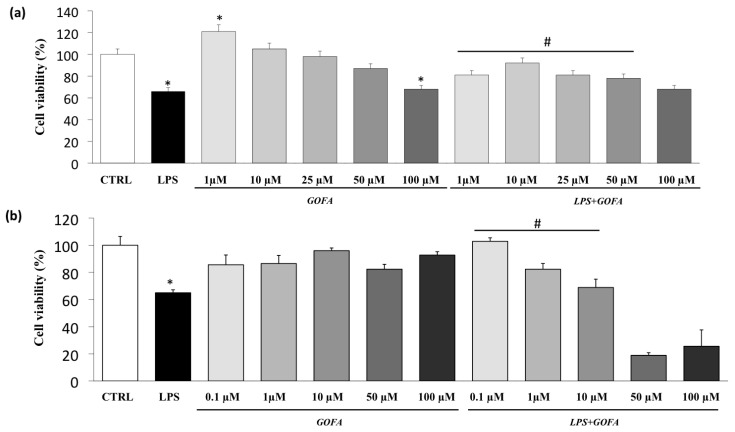
Effects of GOFA on U937 and HCT116 cells viability (MTT test). (**a**) U937 cells were treated with 1, 10, 25, 50, and 100 μM of GOFA for 24 h. Results are shown as percentages of control samples. Data are presented as means ± SD of 3 independent experiments (*n* = 3); (**b**) HCT116 cells were treated with 0.1, 1, 10, 50 and 100 μM of GOFA for 24 h. Data are presented as percentage of control samples (means ± SD, *n* = 3). * *p* < 0.05 vs. control cell; ^#^ *p* < 0.05 vs. cells treated with lipopolysaccharide (LPS) alone.

The viability of U937 and HCT116 cells in the presence of LPS and different concentrations of GOFA was assessed using the MTT assay. The viabilities of U937 and HCT116 cells were reduced by the treatment with LPS (Figure 2). All GOFA concentrations tested, except for 100 µM, did not affect the viability of U937 cells after 24 h of treatment (Figure 2a). However, when the cells were pre-treated with GOFA and then stimulated with LPS, the cell viability in U937 cells was increased when the cells were treated with 1, 10, 25 and 50 µM of GOFA compared to LPS-stimulated cells (Figure 2a). Similarly, all GOFA concentrations tested were not cytotoxic in HCT116 cells (Figure 2b). A significant reduction in cell viability was observed in HCT116 cells pretreated with 50 and 100 µM of GOFA. Thus, the non-cytotoxic GOFA concentrations of 1, 10 and 50 µM were used in the subsequent experiments in U937 cells and 0.1, 1 and 10 µM in HCT116 cells.

### 3.2. GOFA Affects Cell Proliferation and Migration

**Figure 3:**
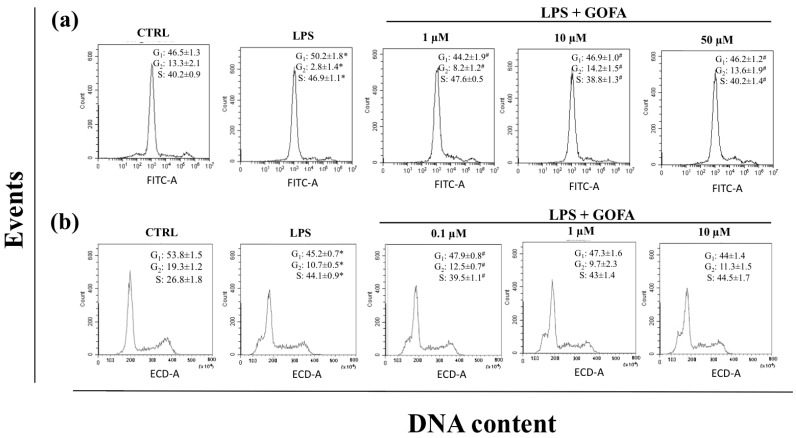
Flow cytometry analysis for cell cycle DNA content in G_0_/G_1_, S, G_2_/M phase after treatment of LPS with and *w/o* GOFA in U937 and HCT116 cells. (**a**) U937 cells were treated with GOFA and the combination of LPS+GOFA and after 24 h cells were stained with propidium iodide for cell cycle analysis and detected by flow cytometry. Percentage of cells was calculated by using MultiCycle software; (**b**) HCT116 cells were treated with GOFA and the combination of LPS+GOFA and after 24 h treatment, cells were processed as described earlier and cell cycle analysis were performed by Flow cytometry. The figures shown are representative analysis from at least three experiments. * *p* < 0.05 vs. control cells; ^#^ *p* < 0.05 vs. cells treated with LPS alone.

We assessed whether GOFA treatment could modulate cell cycle progression in cells stimulated with LPS for 24 h by flow cytometry. As shown in Figure 3, U937 and HCT116 cells presented different cell cycle distributions. In particular, GOFA treatment in LPS-stimulated U937 cells resulted in a significant decrease in cell proliferation compared with the cells stimulated with LPS alone, with progressive and sustained accumulation of cells in the G_2_ phase (Figure 3a). GOFA treatment significantly decreased the percentage of U937 cells in the G_1_ phase as compared to LPS-stimulated cells (from 50.2% to 44.2% (1 µM), 46.9% (10 µM) and 46.2% (50 µM); *p* < 0.001) and increased the percentage of cells in G2/M phase (from 2.8% to 8.2% (1 µM), 14.2% (10 µM) and 13.6% (50 µM); *p* < 0.01). The treatment with GOFA alone did not have an impact on the cell cycle distribution of U937 cells (data not shown).

In HCT116 cells (Figure 3b), the administration of GOFA was slightly effective on cell proliferation compared with the cells stimulated with LPS alone. LPS exposure induced a high increase in the number of cells being in the S phase in comparison with the control sample whereas the percentage of cells both in G_1_ and G_2_/M decreases accordingly. The administration of GOFA together with LPS does not seem able to counteract the effects of the endotoxin, with the exception of the lowest dose (0.1 µM), where the percentage of both G_1_ (from 45.2% to 47.9%; *p* < 0.001) and G_2_/M are increased and the S is decreased (from 44.1% to 39.5%; *p* < 0.01) if compared to the LPS sample, showing then a tendency towards the control sample values.

**Figure 4:**
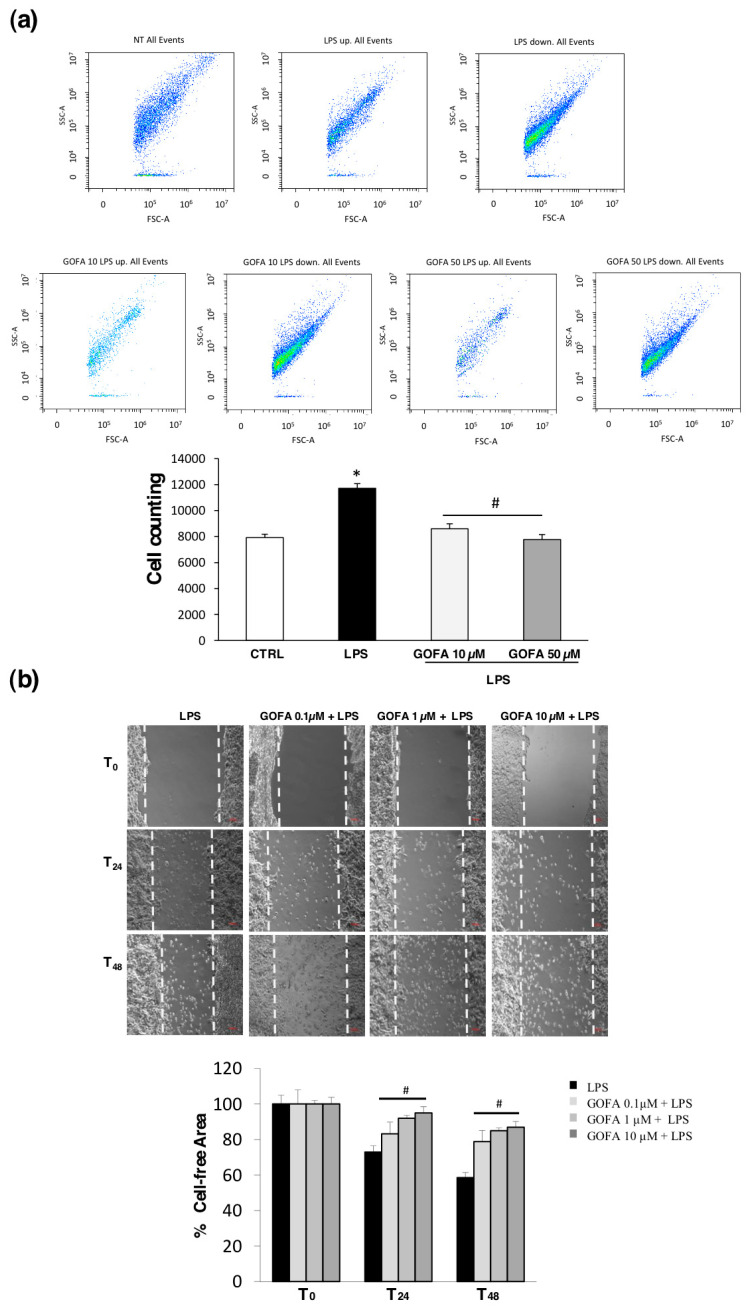
Determination of GOFA effect on U937 and HCT116 cell migration. (**a**) Flow cytometric density dot plots (FSC/SSC) of U937 cells, show that cell populations are consistent in the bottom chamber when cultures are stimulated with LPS and treated with GOFA (10 and 50 µM) for 24 h (upper panel). The numerical representation of the cell flow/min in the different experimental conditions is displayed in the lower panel. As shown, the cell count is significantly augmented after the LPS stimulation. In parallel, the number of cells clearly decreases in the presence of loading concentrations of GOFA. The values are presented as the mean ± SD of three independent experiments. * *p* < 0.05 vs. control cells and # *p* < 0.05 vs. LPS treated cells; (**b**) representative images of scratch assay for HCT116. Cell monolayer was wounded by a 200 µL pipette tip followed by treatment with various concentrations (0.1, 1 and 10 µM) of GOFA for 24 and 48 h after stimulation with LPS. Scale bar = 100 μm (Upper panel). Scratch closure was evaluated measuring the remaining cell-free area and expressed as percentage of the initial cell-free area (lower panel). ^#^ *p* < 0.05 vs. LPS treated cells at the same time.

The effect of GOFA on LPS-induced migration/infiltration of U937 cells was investigated in vitro by using a Boyden chamber Transwell assay. Figure 4a shows representative flow cytometric cell counting results for the samples in the SSC-A vs. FSC-A graphs. The U937 cell count is significantly increased by 32% after the LPS stimulation compared to untreated cells (Figure 4a, * *p* < 0.05). A significant reduction in the cell number is observed in cells pre-treated with GOFA. Specifically, 10 and 50 µM of GOFA have decreased the number of U937 cells by 27% and 34%, respectively (Figure 4a; ^#^ *p* < 0.05) following incubation for 24 h. To measure the HCT116 cell migration ability, we employed a scratch assay (Figure 4b). HCT116 cells were pre-treated for 1 h with GOFA (0.1, 1 and 10 µM) and then stimulated with LPS for 24 and 48 h. A lawn of cells was scratched with a fine tip, and scratch closure size was measured for areas between two layers of scratches. HCT116 cells’ scratch assay revealed that LPS-stimulated cell migration and cell invasion were significantly inhibited by GOFA. Migration cell assays indicated that GOFA have an inhibitory effect on the invasiveness of LPS-treated HCT-116 cells, significantly reducing the matrix invasive potential.

### 3.3. GOFA Attenuates Apoptosis and Cell Senescence 

**Figure 5:**
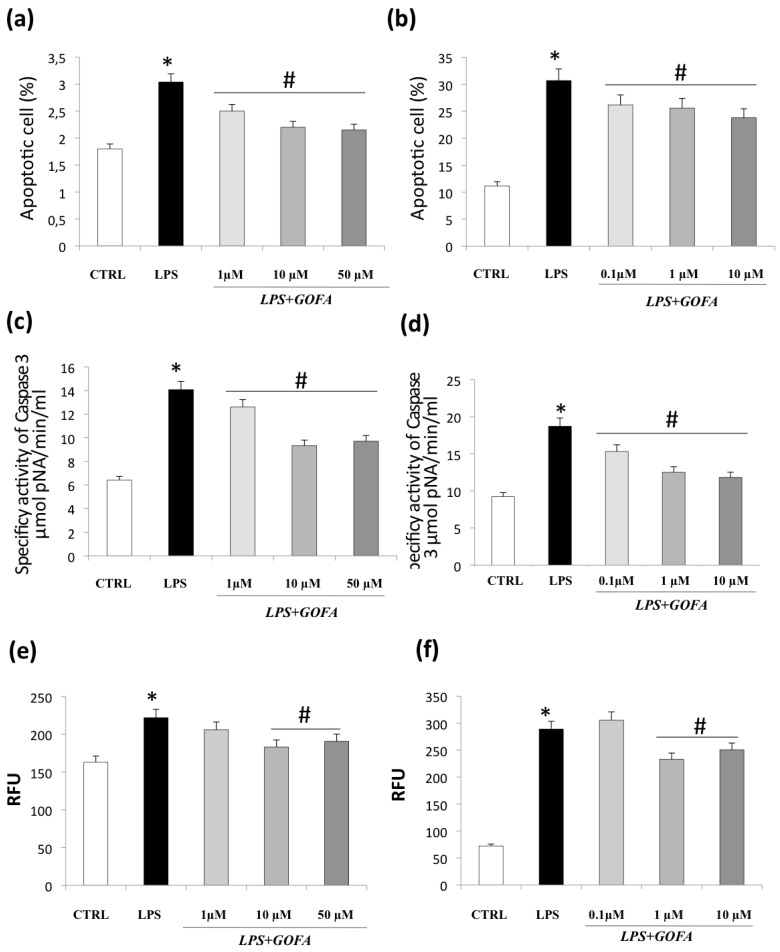
GOFA effects on apoptosis and cell senescence. The effect of GOFA on apoptosis of U937 (**a**) and HCT116 (**b**) cells. The cells were treated with indicated concentration of GOFA for 1 h and then exposed to LPS for 24 h. An Annexin V assay was used for apoptosis detection. (**c**) Bar diagram showing specific effect of GOFA on caspase 3 fluorescent activity on LPS-treated U937 and (**d**) HCT116 cells; (**e**) evaluation of cellular senescence effects of GOFA on U937 and (**f**) HCT116 cells. Cellular senescence was measured using the cellular senescence assay, in which the SA-β-Gal activity was normalized to total protein concentration. All values represent the mean ± SD of three independent experiments. * *p* < 0.05 vs. control cells; ^#^ *p* < 0.05 vs. cell treated with LPS alone.

Since the cell cycle analysis highlights the presence of a subdiploid peak, suggesting the occurrence of apoptosis, a more accurate assay was performed on these cells to investigate the presence of apoptotic cells (Figure 5a,b). Flow cytometry analysis performed on U937 cells (Figure 5a), where the subdiploid peak was observed just in two experimental conditions, showed that the treatment with LPS determined a significant increase in the apoptotic rate. The effects of GOFA pre-treatment on the monocyte cell line turned out to be effective even at a lower concentration.

The same assay confirmed the presence of apoptosis in all the HCT116 samples (Figure 5b), mainly in the cells exposed to LPS. The percentage of apoptotic cells is reduced when the GOFA is added to the culture medium in what appears to be a dose dependent manner.

In line with apoptosis data, caspase 3 activity, known to be crucial mediators of programmed cell death, showed a marked increase in LPS-stimulation in both cell lines as compared to control (Figure 5c,d). Pre-treatment with all GOFA concentrations used significantly reduced the percentage of apoptotic cells.

Furthermore, since there is increasing evidence that the senescence response could have a detrimental effect owing to the complexity of the secretory senescent phenotype [30], we investigated whether GOFA could affect the cell senescence. We investigated senescence via a β-galactosidase activity assay (Figure 5e,f). The results demonstrate that LPS stimulation induced a significantly higher proportion of SA-β-gal-positive in both cell lines compared with the control (panel e and f). The pre-treatment with GOFA determined a reduction in the total cell population with β-gal-positive senescent cells, although the reduction was significantly greater at higher concentrations for both cell lines. These results show that GOFA could act as a senolytic compound in a dose-dependent manner in inflammatory/stimulated cells.

### 3.4. GOFA Effects on MMP-9 Expression and Activity 

**Figure 6:**
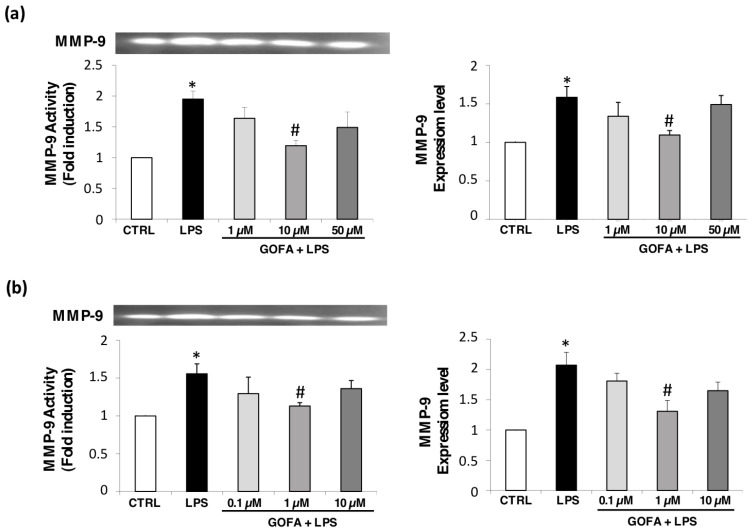
GOFA effects on MMP-9 for U937 (**a**) and HCT-116 (**b**) cell lines. Cell-free conditioned media were assayed for MMP-2 and MMP-9 activity by gelatin zymography. The activity of MMP-9 (92 kDa) (bottom) was reported with fold induction values compared to control MMP-9 activity. Zymogram (top) is representative of 6 gels using 3 separate pools of total protein extracted from cell lines. Changes in MMP-9 gene expression were determined by qRT-PCR assay, using the (ΔΔCt) method and 18S as housekeeping gene. One-way ANOVA, values represent mean ± SD (*n* = 6); ^*^ *p* < 0.05 vs. control cells; ^#^ *p* < 0.05 vs. cell treated with LPS alone.

Previous studies have evidenced that MMPs, particularly MMP-2 and -9, play important roles in cancer metastasis, contributing to angiogenesis, intravasation of tumor cells, and cell migration and invasion [14]. To investigate whether GOFA modulates MMP-9 expression and activity, we performed qRT-PCR and zymography analysis following GOFA treatment on U937 and HCT116 cells, after inducing with LPS (Figure 6). The levels of MMP-9 were not modified by GOFA treatment alone (data not shown). The MMP-9 activity increased by 34% and 35.78% after 24 h, respectively, in U937 and HCT116 cells (* *p* < 0.05 for both) after LPS-stimulation compared to control cells (Figure 6). In contrast, MMP-9 activity was significantly decreased by 30.95% in U937 cells pre-treated with 10 µM of GOFA and by 27.36% in HCT 116 following the pre-treatment with 1 µM of GOFA, when compared to LPS-treated cells (^#^ *p* < 0.05 for both). MMP-9 expression data performed by qRT-PCR showed a similar trend of activity. In summary, our data indicate that GOFA-attenuated invasion of LPS-stimulated U937 and HCT116 cells was mediated through the downregulation of MMP-9 at the transcriptional level.

### 3.5. GOFA Inhibits Invasion of U937 and HCT116 Cells by Reducing MMP-9 Activity via ROS/ERK Pathway

**Figure 7:**
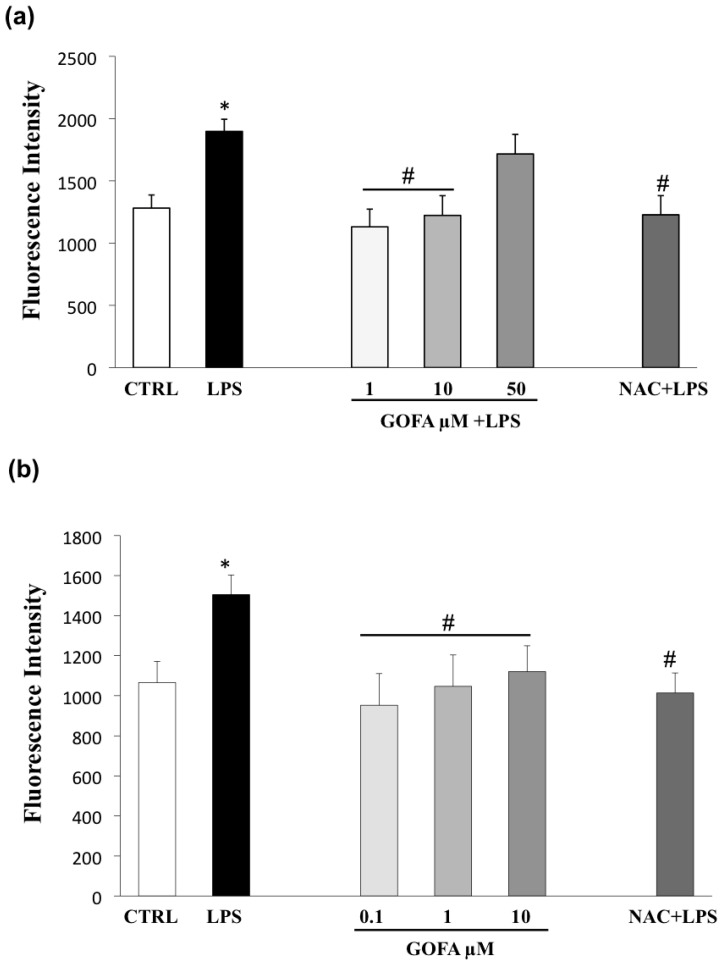
Effects of GOFA on preventing LPS-induced reactive oxygen species (ROS) production in U937 (**a**) and HCT116 (**b**) cells. Antioxidant activity of GOFA against oxidative stress LPS-induced, measured by DCFH-DA assay and compared to ROS scavenger, N-acetyl-L-cysteine (NAC). Results (*n* = 3) are reported as means ± SD. ^*^ *p* < 0.05 vs. control cells; ^#^ *p* < 0.05 vs. cell treated with LPS alone.

**Figure 8:**
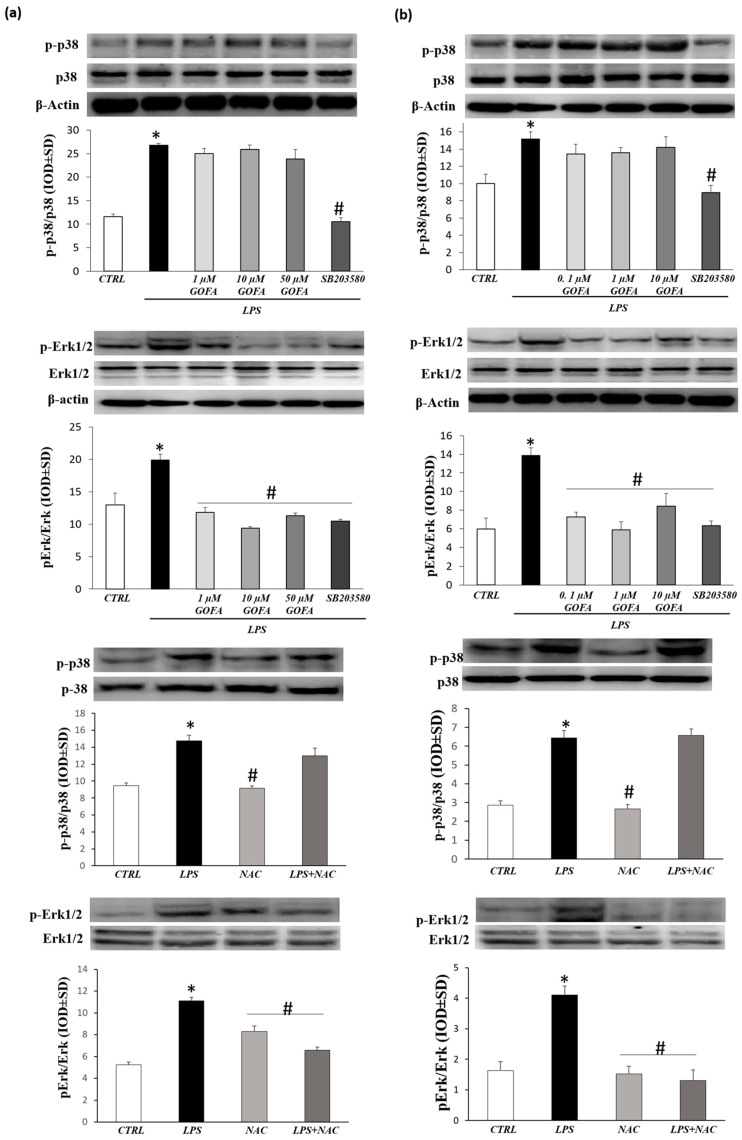
Effects of GOFA on p-p38 and pERK1/2 kinase. Analysis of phosphorylation level of p38 and extracellular signal-regulated kinases (ERK) in GOFA-treated U937 (**a**) and HCT116 (**b**) by Western blotting. Selective inhibitor of p38 (SB203580), ERK (PD980559) and ROS (N-acetyl-L-cysteine, NAC) was used as positive control. Cells were pretreated with selective inhibitors for 30 min and treated with LPS or GOFA + LPS for 24 h. The ratios of p-p38/β-actin and p-ERK/β-actin were calculated based on the relative band densities, respectively. These data are the results of a typical experiment (*n* = 3); ^*^ *p* < 0.05 vs. control cells; ^#^ *p* < 0.05 vs. cells treated with LPS alone.

**Figure 9:**
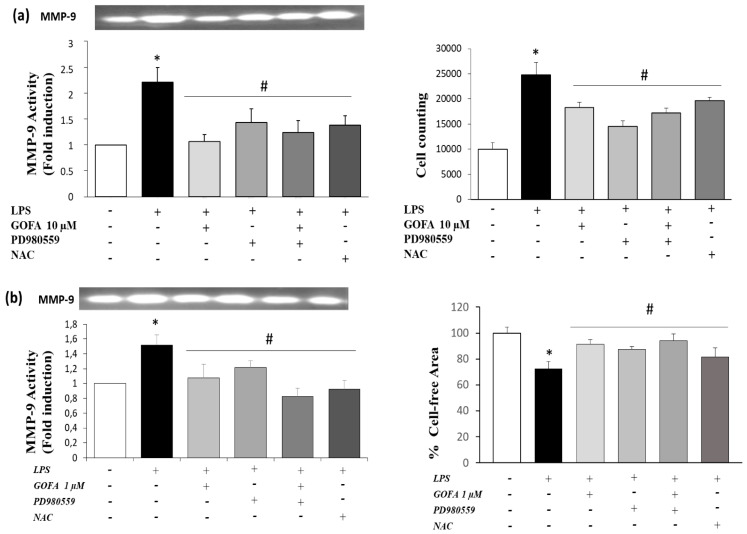
Effects of pharmacological inhibitors on MMP-9 activation and migration in U937 (A) and HCT-116 (B) cell lines. Cells were treated or not with GOFA, selective inhibitor of ERK (PD980559) and ROS (NAC). (**a**) MMP-9 activity by zymography (left) and numerical representation of the cell flow/min (right) in the different experimental conditions of U937 cells. ^*^ *p* < 0.05 vs. control cells; ^#^ *p* < 0.05 vs. LPS treated cells Data are reported as fold induction values (mean ± SD, *n* = 6). * *p* < 0.05 vs. cells treated with LPS alone. (**b**) MMP-9 activity by zymography (left) and histogram bar of scratch closure measuring the remaining cell-free area expressed as percentage of the initial cell-free area (right). * *p* < 0.05 vs. control cells (T_0_); ^#^ *p* < 0.05 vs. cells treated with LPS alone. The values are presented as the mean ± SD of three independent experiments.

The mitogen-activated protein kinase (MAPK) signaling pathway has a pivotal role in cancer events and its target molecules (p38 and ERK) are linked to tumor growth, migration, and invasion [14]. Moreover, as many studies have reported that MAPK signaling activation is downstream of redox imbalance, we assessed the level of intracellular ROS by flow cytometric analysis with DCFH-DA. Figure 7 shows an increase in ROS generation in U937 and HCT116 cells treated with LPS for 24 h. Noticeably, pre-treatment with GOFA in LPS stimulated cell lines abrogated significantly ROS generation, at all concentrations examined, in both U937 and HCT116 cells with a comparable entity elicited with N-acetyl-L-cysteine (NAC) (the ROS scavenger) pretreatment. To better characterize the mechanism underlying the anti-proliferative effect of GOFA, we analyzed whether it influenced p38 and ERK expression. To inspect the involvement of ERK and p38 signaling pathways in GOFA mediated effect, LPS-stimulated U937 and HCT116 cells, were exposed to GOFA, in the presence of p38 and pERK1/2 inhibitors. LPS activated p38 and ERK signaling pathways, as evidenced by their increased phosphorylation, in both cell lines (Figure 8). GOFA did not affect the phosphorylation of p38 in both cell lines compared to LPS treatment with or without p38 inhibitor, used as a positive control (Figure 8). The result of expression analysis of pERK shows that GOFA downregulates LPS-induced phosphorylation of ERK in both cell lines, acting like a pERK inhibitor. A greater significance was obtained for GOFA concentration of 10 and 1 µM on U937 and HCT116, respectively. Thus, to perform subsequent experiments, these GOFA concentrations were used. LPS is well-known to activate several genes, many of which are mediated by the MAPKs. As LPS have an effect on the cellular redox state, we attempted to define, in this in vitro study, whether there is a differential effect of NAC on LPS activation of the MAPKs, ERK1/2 and p38. However, whereas NAC inhibited LPS-induced ERK1/2 phosphorylation, there was no such inhibitory effect on LPS-induced p38 phosphorylation in both cell lines (Figure 8). Testing the hypothesis that GOFA acts via ERK signaling pathways, LPS-activated U937 and HCT116 cells and pre-treated with GOFA, were analyzed in the presence of specific inhibitor of ERK1/2 (PD98059) and the activity of MMP-9 was performed (Figure 9). The ROS scavenger, NAC, was used as a negative control for the evaluation of the effect of ROS in inducing MMP-9.

In U937 and HCT116 cells LPS-activated and pre-treated with GOFA, pERK1/2 inhibition significantly attenuate the activity of MMP-9. Remarkably, treatment with GOFA in LPS stimulated cell lines significantly abrogated MMP-9 activity with a similar entity caused by ROS scavenger pretreatment, highlighting a greater significance on the monocytic cells compared to the colon rectal cancer cell. These results indicate that the pERK1/2 signaling pathway is involved in GOFA modulation of MMP-9 in monocytic and colorectal cancer cells LPS-induced.

Finally, the effect of ERK inhibitors on cellular migration after 24 h of GOFA treatment was tested (Figure 9) in order to further verify whether the ROS/ERK signaling acts as upstream signaling pathways in GOFA-observed anti-proliferative effects on U937 and HCT116 cells. The inhibition of ERK kinase resulted in decreased GOFA-reduced cell migration of both cells types compared to LPSactivated cells. Cells treated with GOFA produce the same effect as NAC on inhibiting LPS-induced proliferation. These results confirm that GOFA reduces the activation and expression of MMP-9 and cell migration, affecting ROS/pERK signaling pathways in U937 and HCT116 cells.

## 4. Discussion

The main objective of this research was to investigate the ability of GOFA to affect the migration and invasion of two different cell lines, monocytic human myeloid leukemia and colon cancer cells, using a well-known migration assay in vitro model.

We valuated whether GOFA has the properties to attenuate LPS-promoted malignancy on U937 and HCT116 cell lines through the modulation of proliferative/migration activity and apoptosis/senescence and to determine, at least in part, the molecular mechanisms underlying GOFA’s effects.

Nowadays, the research and the development of anti-proliferative/invasive natural chemical compounds for tumor cells are under active investigation, due to the toxic and drug resistance side effects of the current chemotherapy drugs.

In the last few years, many oxyprenylated natural phenylpropanoids displayed either cytostatic or cytotoxic growth inhibitory effects in vitro [23]. Miyamoto S. et al. (2008) reported that 3-(4-geranyloxy-3-methoxyphenyl)-2-trans-propenoyl-L-alanyl-L-proline (GAP), a derivative of ferulic acid, inhibited Azoxymethane (AOM)/Dextran Sodium Sulfate (DSS)-induced, colitis-related, colonic carcinogenesis through the modulation of cell proliferation, the reduction in inflammation, and the suppression of oxidative damage, enhancing the antioxidant enzyme HO-1, without any adverse effects in mice [31].

GOFA is an oxyprenylated natural product that has been reported to show interesting and promising therapeutic potentialities, including chemopreventive effects in a variety of tissues and anti-inflammatory activity through oxidative stress inhibition [32,33].

Recently, we demonstrated that GOFA permeates the cell membrane and accumulates intracellularly for a relatively long period [34]. These pharmacokinetic data are crucial for explain its therapeutic efficiency and to understand the exact molecular mechanism underlying these unclear effects.

Chronic inflammation and genetic-cellular signaling modification contribute to the development and progression of cancer. Developing evidence indicates the mutual interdependence of local immune response and systemic inflammation in the progression of malignant diseases. This acquisition provides a therapeutic opportunity to target intracellular inflammatory signaling pathways to impair the progression from chronic inflammation to malignant disease [35].

Toll-like receptor 4 (TLR4)-mediated inflammatory signaling, known to recognize LPS, was described as a key player in the relationship with the malignant behavior of cancer cells.

TLR4 is generally reported in immune cells but aberrant expression was found in various types of carcinoma, showing a correlation between its expression level and the malignancy of cancer. TLR4 could modulate immunosuppressive cytokine induction and apoptosis resistance, events responsible of cancer cells’ immune escape [36].

LPS is a well-known inflammatory stimulus and has been reported to stimulate metastasis in human colorectal carcinoma cell lines (SW48) by increasing migration, adhesion, and invasion in a TLR4-dependent way [37].

Consequently, this present study aimed to address whether the suppression of LPS-mediate TLR4-signaling reduce inflammation-induced cancer progression in addition to dampen the inflammatory response, as we reported previously [38]. Our data show that GOFA inhibits LPS-induced cell proliferation and migration/invasion in U937 and HCT116 cells.

We observed a significant reduction in the proliferation and migration of GOFA-pretreated cells using a scratch assay for HCT116 cells and a modified Boyden chambers for U937 cells.

These results are consistent with previous data that described anti-proliferative effects of GOFA in colitis-related colon carcinogenesis in vivo, via regulating the activity of the NF-κB-mediated pathway and the expression of proinflammatory cytokines [33].

Tumorigenesis and metastatic processes are complex in which two important cellular events, such as cellular senescence and apoptosis, are compromised during tumorigenesis and metastatic [39,40].

In the present study, we found, for the first time, that in two different cell-lines, GOFA caused a dose-dependent reduction in the percentages of SA-β-gal-positive cells in response to LPS, indicating an impairment of the senescence process. Moreover, GOFA caused a dose-dependent reduction in apoptosis, as assessed by Annexin V/PI staining. Furthermore, GOFA suppressed cellular growth in LPS-stimulated HCT116 and U937 cells as indicated by the arrest of cell cycle distribution. The lack of effect observed with low GOFA concentrations might be attributed to the short duration of treatment.

In this case, the potential senolytic efficacy of natural bioactive compounds on LPS-induced senescent-associated secretory phenotype (SASP) might be crucial. It is an emerging concept that senescent cells could display an increased metabolic activity and develop the SASP, which includes several metabolites, such as pro-inflammatory cytokines and metalloproteinases involved in inflammatory processes, stimulation of cell growth and survival of nearby malignant cells [41].

MMP are extracellular proteases representing a potential therapeutic target for their role in inflammatory processes and tumor progression. The proteolytic degradation of the extracellular matrix (ECM) by MMPs is required for migration and invasion of malignant cancer cells [42,43].

Among the various MMPs described and involved in malignant cancer cells, our attention has focused on gelatinase MMP-9, considered as the “prognostic biomarkers” for different malignant tumors. Firstly, MMP-9 is overexpressed in many experimental animal models and different histological types of human malignant tumors (solid cancers such as colorectal and hematological malignancies), and secondly, its expression and activity is often associated with greater tumor aggression and an unfavorable prognosis [44,45].

Our results show that LPS stimulation caused upregulated MMP-9 expression and activity in both U937 and HCT116 cell lines. GOFA significantly inhibited MMP-9 enzymatic activity and expression. These results indicate that the anti-migration and anti-invasion effect of GOFA may be mediated by the inhibition of MMP-9 enzyme activity and expression.

Numerous reports have suggested that MMP-9 expression/activity regulation and tumor development and progression are critically mediated by MAPK signaling pathway.

LPS-induced MMP-9 expression and activity involves the activation of the ERK1/2 and p38 signaling pathways [1,46,47].

Hence, to investigate the molecular mechanism underlying GOFA-reduced MMP-9 expression in monocytic and colorectal cancer cells, we assessed the effects of specific inhibitors of MAPKs (ERK and p38) on LPS-induced migration and MMP-9 activity. Moreover, since ROS formation mediates a wide range of cellular processes, including metastasis by triggering the MAPK activation, we examined the effect of GOFA on ROS generation.

This study has shown that LPS increased MMP-9 expression and activation by phosphorylation of p38 and ERK and by ROS accumulation in both model cells.

Among MAPKs, ERK phosphorylation has been shown to be significantly downregulated by the GOFA treatment in a dose-dependent manner in both HCT116 and U937 cells.

The experiment with ERK1/2 inhibitor (PD98059) revealed that GOFA reduces LPS-induced invasion and MMP-9 activity by ERK1/2 activation through ROS generation.

Taken together, these data show that GOFA could represent a potential natural anticancer molecule that inhibits HCT116 and U937 cell proliferation and migration in combination to apoptosis and senescence attenuation. The reduction in MMP-9 enzyme activity and gene expression, associated with the inhibition of pERK and p38 activity/expression correlated with ROS levels, accounts for the anti-metastatic effects of GOFA, as previously reported in in vivo studies [31].

These new findings on the role of GOFA in inhibiting cancer cell proliferation and migration could be therapeutically beneficial to prevent tumor metastasis.

## References

[1] Patruno A., Ferrone A., Costantini E., Franceschelli S., Pesce M., Speranza L., Amerio P., D’Angelo C., Felaco M., Grilli A. (2018). Extremely low-frequency electromagnetic fields accelerates wound healing modulating MMP-9 and inflammatory cytokines. Cell Prolif..

[2] Jeong D., Lee J., Park S.H., Kim Y.A., Park B.J., Oh J., Sung G.H., Aravinthan A., Kim J.H., Kang H. (2019). Antiphotoaging and Antimelanogenic Effects of Penthorum chinense Pursh Ethanol Extract due to Antioxidant- and Autophagy-Inducing Properties. Oxid. Med. Cell Longev..

[3] Bauvois B. (2012). New facets of matrix metalloproteinases MMP-2 and MMP-9 as cell surface transducers: Outside-in signaling and relationship to tumor progression. Biochim Biophys Acta..

[4] Franceschelli S., Pesce M., Ferrone A., Gatta D.M., Patruno A., De Lutiis M.A., Quiles J.L., Grilli A., Felaco M., Speranza L. (2017). Biological Effect of Licochalcone C on the Regulation of PI3K/Akt/eNOS and NF-κB/iNOS/NO Signaling Pathways in H9c2 Cells in Response to LPS Stimulation. Int. J. Mol. Sci..

[5] Pesce M., Speranza L., Franceschelli S., Ialenti V., Patruno A., Febo M.A., De Lutiis M.A., Felaco M., Grilli A. (2011). Biological role of interleukin-1β in defensive aggressive behavior. J. Biol. Regul. Homeost. Agents.

[6] Pesce M., Patruno A., Speranza L., Reale M. (2013). Extremely low frequency electromagnetic field and wound healing: Implication of cytokines as biological mediators. Eur. Cytokine Netw..

[7] Mittal M., Siddiqui M.R., Tran K., Reddy S.P., Malik A.B. (2014). Reactive oxygen species in inflammation and tissue injury. Antioxid. Redox Signal..

[8] Iarlori C., Gambi D., Lugaresi A., Patruno A., Felaco M., Salvatore M., Speranza L., Reale M. (2008). Reduction of free radicals in multiple sclerosis: Effect of glatiramer acetate (Copaxone). Mult. Scler..

[9] Bulboaca A.E., Boarescu P.M., Porfire A.S., Dogaru G., Barbalata C., Valeanu M., Munteanu C., Râjnoveanu R.M., Nicula C.A., Stanescu I.C. (2020). The Effect of Nano-Epigallocatechin-Gallate on Oxidative Stress and Matrix Metalloproteinases in Experimental Diabetes Mellitus. Antioxidants.

[10] Bonnans C., Chou J., Werb Z. (2014). Remodelling the extracellular matrix in development and disease. Nat. Rev. Mol. Cell Biol..

[11] Tallant C., Marrero A., Gomis-Rüth F.X. (2010). Matrix metalloproteinases: Fold and function of their catalytic domains. Biochim. Biophys. Acta.

[12] Manicone A.M., McGuire J.K. (2008). Matrix metalloproteinases as modulators of inflammation. Semin. Cell Dev. Biol..

[13] Tokito A., Jougasaki M. (2016). Matrix Metalloproteinases in Non-Neoplastic Disorders. Int. J. Mol. Sci..

[14] Kessenbrock K., Plaks V., Werb Z. (2010). Matrix metalloproteinases: Regulators of the tumor microenvironment. Cell.

[15] Pesce M., Franceschelli S., Ferrone A., De Lutiis M.A., Patruno A., Grilli A., Felaco M., Speranza L. (2015). Verbascoside down-regulates some pro-inflammatory signal transduction pathways by increasing the activity of tyrosine phosphatase SHP-1 in the U937 cell line. J. Cell Mol. Med..

[16] Epifano F., Fiorito S., Taddeo V.A., Genovese S. (2015). 4′-Geranyloxyferulic acid: An overview of its potentialities as an anti-cancer and anti-inflammatory agent. Phytochem. Rev..

[17] Perera P.Y., Mayadas T.N., Takeuchi O., Akira S., Zaks-Zilberman M., Goyert S.M., Vogel S.N. (2001). CD11b/CD18 acts in concert with CD14 and Toll-like receptor (TLR) 4 to elicit full lipopolysaccharide and taxol-inducible gene expression. J. Immunol..

[18] Kojima M., Morisaki T., Izuhara K., Uchiyama A., Matsunari Y., Katano M., Tanaka M. (2000). Lipopolysaccharide increases cyclo-oxygenase-2 in a colon carcinoma cell line through nuclear factor-kB activation. Oncogene.

[19] Chow J.C., Young D.W., Golenbock D.T., Christ W.J., Gusovsky F. (1999). Toll-like receptor-4 mediates lipopolysaccharide-induced signal transduction. J. Biol. Chem..

[20] Ikebe M., Kitaura Y., Nakamura M., Tanaka H., Yamasaki A., Nagai S., Wada J., Yanai K., Koga K., Sato N. (2009). Lipopolysaccharide (LPS) increases the invasive ability of pancreatic cancer cells through the TLR4/MyD88 signaling pathway. J. Surg. Oncol..

[21] Franceschelli S., Gatta D.M.P., Pesce M., Ferrone A., Quiles J.L., Genovese S., Epifano F., Fiorito S., Taddeo V.A., Patruno A. (2016). New approach in translational medicine: Effects of electrolyzed reduced water (ERW) on NF-κB/iNOS pathway in U937 cell line under Altered Redox State. Int. J. Mol. Sci..

[22] (2019). Modulation of CAT-2B-Mediated l-Arginine Uptake and Nitric Oxide Biosynthesis in HCT116 Cell Line Through Biological Activity of 4′-Geranyloxyferulic Acid Extract from Quinoa Seeds. Int. J. Mol. Sci..

[23] Bruyere C., Genovese S., Lallemand B., Ionescu-Motatu A., Curini M., Kiss R., Epifano F. (2011). Growth inhibitory activities of oxyprenylated and non-prenylated naturally occurring phenylpropanoids in cancer cell lines. Bioorg. Med. Chem. Lett..

[24] Patruno A., Fornasari E., Di Stefano A., Cerasa L.S., Marinelli L., Baldassarre L., Sozio P., Turkez H., Franceschelli S., Ferrone A. (2015). Synthesis of a novel cyclic prodrug of S-allyl-glutathione able to attenuate LPS-induced ROS production through the inhibition of MAPK pathways in U937 cells. Mol. Pharm..

[25] Patruno A., Pesce M., Marrone A., Speranza L., Grilli A., De Lutiis M.A., Felaco M., Reale M. (2012). Activity of matrix metallo proteinases (MMPs) and the tissue inhibitor of MMP (TIMP)-1 in electromagnetic field-exposed THP-1 cells. J. Cell Physiol..

[26] Patruno A., Franceschelli S., Pesce M., Maccallini C., Fantacuzzi M., Speranza L., Ferrone A., De Lutiis M.A., Ricciotti E., Amoroso R. (2012). Novel aminobenzyl-acetamidine derivative modulate the differential regulation of NOSs in LPS induced inflammatory response: Role of PI3K/Akt pathway. Biochim. Biophys. Acta.

[27] Grilli A., De Lutiis M.A., Patruno A., Speranza L., Cataldi A., Centurione L., Taccardi A.A., Di Napoli P., De Caterina R., Barbacane R. (2003). Effect of chronic hypoxia on inducible nitric oxide synthase expression in rat myocardial tissue. Exp. Biol. Med..

[28] Cacciatore I., Marinelli L., Di Stefano A., Di Marco V., Orlando G., Gabriele M., Gatta D.M.P., Ferrone A., Franceschelli S., Speranza L. (2018). Chelating and antioxidant properties of l-Dopa containing tetrapeptide for the treatment of neurodegenerative diseases. Neuropeptides.

[29] Di Nisio C., Sancilio S., Di Giacomo V., Rapino M., Sancillo L., Genovesi D., Di Siena A., Rana R.A., Cataldi A., Di Pietro R. (2016). Involvement of cyclic-nucleotide response element-binding family members in the radiation response of Ramos B lymphoma cells. Int. J. Oncol..

[30] Campisi J. (2011). Cellular senescence: Putting the paradoxes in perspective. Curr. Opin. Genet. Dev..

[31] Miyamoto S., Epifano F., Curini M., Genovese S., Kim M., Ishigamori-Suzuki R., Yasui Y., Sugie S., Tanaka T. (2008). A novel prodrug of 4′-geranyloxy-ferulic acid suppresses colitis-related colon carcinogenesis in mice. Nutr. Cancer.

[32] Genovese S., Epifano F. (2012). Recent developments in the pharmacological properties of 4′-geranyloxyferulic acid, a colon cancer chemopreventive agent of natural origin. Curr. Drug Targets.

[33] Tanaka T., de Azevedo M.B., Durán N., Alderete J.B., Epifano F., Genovese S., Tanaka M., Tanaka T., Curini M. (2010). Colorectal cancer chemoprevention by 2 beta-cyclodextrin inclusion compounds of auraptene and 4′-geranyloxyferulic acid. Int. J. Cancer.

[34] Taddeo V.A., Genovese S., Carlucci G., Ferrone V., Patruno A., Ferrone A., de Medina P., Fiorito S., Epifano F. (2017). Quantitative profiling of 4′-geranyloxyferulic acid and its conjugate with l-nitroarginine methyl ester in mononuclear cells by high-performance liquid chromatography with fluorescence detection. J. Pharm. Biomed. Anal..

[35] Krivennikov S.I., Greten F.R., Karin M. (2010). Immunity, inflammation, and cancer. Cell.

[36] Chen C.Y., Kao C.L., Liu C.M. (2018). The Cancer Prevention, Anti-Inflammatory and Anti-Oxidation of Bioactive Phytochemicals Targeting the TLR4 Signaling Pathway. Int. J. Mol. Sci..

[37] Zhang R., Qi F., Zhao F., Li G., Shao S., Zhang X., Yuan L., Feng Y. (2019). Cancer-associated fibroblasts enhance tumor-associated macrophages enrichment and suppress NK cells function in colorectal cancer. Cell Death Dis..

[38] Genovese S., Fiorito S., Taddeo V.A., Epifano F., Paciotti R., Coletti C., Franceschelli S., Speranza L., Ferrone A., Felaco M. (2016). Effects of geranyloxycinnamic acids on COX-2 and iNOS functionalities in LPS-stimulated U937 mononuclear cells. Chem. Sel..

[39] Baker D.J., Alimirah F., van Deursen J.M., Campisi J., Hildesheim J. (2017). Oncogenic senescence: A multi-functional perspective. Oncotarget.

[40] Cerella C., Grandjenette C., Dicato M., Diederich M. (2016). Roles of Apoptosis and Cellular Senescence in Cancer and Aging. Curr. Drug Targets.

[41] Coppé J.P., Desprez P.Y., Krtolica A., Campisi J. (2010). The senescence-associated secretory phenotype: The dark side of tumor suppression. Annu. Rev. Pathol..

[42] Klein T., Bischoff R. (2011). Physiology and pathophysiology of matrix metalloproteases. Amino Acids.

[43] Roy R., Yang J., Moses M.A. (2009). Matrix metalloproteinases as novel biomarkers and potential therapeutic targets in human cancer. J. Clin. Oncol..

[44] Turpeenniemi-Hujanen T. (2005). Gelatinases (MMP-2 and -9) and their natural inhibitors as prognostic indicators in solid cancers. Biochimie.

[45] Yu X.F., Han Z.C. (2006). Matrix metalloproteinases in bone marrow: Roles of gelatinases in physiological hematopoiesis and hematopoietic malignancies. Histol. Histopathol..

[46] Lee W.J., Shin C.Y., Yoo B.K., Ryu J.R., Choi E.Y., Cheong J.H., Ryu J.H., Ko K.H. (2003). Induction of matrix metalloproteinase-9 (MMP-9) in lipopolysaccharidestimulated primary astrocytes is mediated by extracellular signal-regulated protein kinase 1/2 (Erk1/2). Glia.

[47] Underwood D.C., Osborn R.R., Bochnowicz S., Webb E.F., Rieman D.J., Lee J.C., Romanic A.M., Adams J.L., Hay D.W., Griswold D.E. (2000). SB 239063, a p38 MAPK inhibitor, reduces neutrophilia, inflammatory cytokines, MMP-9, and fibrosis in lung. Am. J. Physiol. Lung Cell Mol. Physiol..

